# An Exploratory Semantic Analysis of Age-Related Stereotypes in OpenAI’s GPT 4o Model

**DOI:** 10.1093/geroni/igaf122.1471

**Published:** 2025-12-31

**Authors:** Wan Hong, Moon Choi

**Affiliations:** Korea Advanced Institute of Science & Technology, Daejeon, Republic of Korea; Korea Advanced Institute of Science & Technology, Daejeon, Republic of Korea

## Abstract

Generative artificial intelligence, particularly large language models (LLMs), has become a powerful tool for navigating information, potentially shaping users’ ways of thinking and influencing their perceptions of different social groups. While a growing body of research has examined the presence of racism and sexism in LLM-generated content, the issue of ageism remains relatively underexplored. This study investigates age-related stereotypes in texts generated by LLMs utilizing natural language processing (NLP) techniques. To ensure methodological rigor, extensive pilot testing was conducted to develop a neutral prompt that did not fish for bias but still generated coherent responses. The final prompt followed the structure: “Describe the personality of a [AGE]-year-old person.” Text responses were collected using OpenAI’s GPT-4o API in February 2025, with the AGE variable ranging from 10 to 90 in increments of 10. The analysis was guided by the Stereotype Content Model, which evaluates social cognition along two key dimensions: warmth (sociability, morality) and competence (ability, agency). Findings indicate that descriptions of individuals aged 60 and older exhibit relatively lower competence than those of younger individuals. These findings suggest that OpenAI’s GPT 4o model might embed age-related stereotypes, even when using mostly positive terms, potentially causing their users to be repeatedly exposed to characterizations containing these stereotypes. Future research should explore the underlying mechanisms driving age-related stereotypes in LLMs and develop strategies to mitigate them, ensuring more age-inclusive AI-generated content.

